# Ten-Year Stability of Clinical Attachment after Regenerative Treatment of Infrabony Defects and Controls

**DOI:** 10.3390/jcm11030543

**Published:** 2022-01-21

**Authors:** Hari Petsos, Ilona Koronna, Tatjana Ramich, Katrin Nickles, Bettina Dannewitz, Beate Schacher, Peter Eickholz

**Affiliations:** 1 Department of Periodontology, Center of Dentistry and Oral Medicine (Carolinum), Johann Wolfgang Goethe-University Frankfurt/Main, Theodor-Stern-Kai 7, 60596 Frankfurt, Germany; koronna@gmx.de (I.K.); tatjanaramich@yahoo.de (T.R.); nickles@med.uni-frankfurt.de (K.N.); dannewitz@med.uni-frankfurt.de (B.D.); schacher@em.uni-frankfurt.de (B.S.); eickholz@med.uni-frankfurt.de (P.E.); 2 Private Practice, Westenhellweg 10, 59494 Soest, Germany; 3 Private Practice, Salzstraße 21, 63450 Hanau, Germany; 4 Private Practice, Talstrasse 1A, 68259 Mannheim, Germany; 5 Private Practice, Langgasse 36-38, 35781 Weilburg, Germany

**Keywords:** enamel matrix derivatives, guided tissue regeneration, attachment loss, tooth loss, periodontitis, surgical procedures

## Abstract

Background: A similar long-term stable clinical attachment level (CAL) of infrabony defects (IBDs) after regenerative treatment compared to control teeth would indicate a high level of stability resulting from the regenerative approach. Methods: Patients with a regeneratively treated IBD were screened 120 ± 12 months postoperatively for eligibility for study participation, and were included if complete baseline and 12-month examinations (plaque (PlI), periodontal probing depth (PPD), CAL) were available and a respective control tooth could be identified. Re-examination included clinical examination (PPD, CAL, PlI/GI, bleeding on probing, plaque control record, gingival bleeding index). Results: A total of 27 patients (16 females; age (median; lower/upper quartile): 57.0; 44.0/60.0 years; 6 smokers) contributed 27 IBDs (test), for each of which a control tooth was identified. Five test teeth (18.5%) were lost between 12 and 120 months. The remaining 22 test teeth revealed a significant CAL gain after 1 (2.5 mm; 1.0/4.0 mm, *p* < 0.0001) and 10 (2.5 mm; 0.5/3.5 mm, *p* < 0.0001) years, whereas control teeth were stable (1 year: 0.0 mm; 0.0/1.0 mm, *p* = 0.396; 10 years: 0.0 mm; −1.0/1.5 mm, *p* = 0.215). The study did not detect any significant CAL change between 1 and 10 years for test (−0.5 mm; −1.0/0.5 mm, *p* = 0.414) and control teeth (0.0 mm; −1.0/1.0 mm, *p* = 0.739). In 15 patients, test and control teeth revealed stable CAL values between 12 and 120 months. Conclusion: Regenerative treatment of IBDs exhibited stability comparable to non-surgically treated, periodontally reduced sites over a 10-year period.

## 1. Introduction

Early pioneering studies provided histological evidence that it is possible to regenerate lost periodontal attachment [1]. However, since histology plays a subordinate role in everyday clinical practice, numerous studies and systematic reviews followed, showing that regenerative therapy for infrabony defects (IBDs) is superior to conventional open-flap debridement (OFD) [2,3,4,5,6,7,8]. Not least for this reason, the treatment of IBDs of more than 3 mm was approved with a strong recommendation in the EFP (European Federation of Periodontology) S3 level clinical practice guidelines [9]. However, since the regenerative procedure is both a surgical and cost-intensive procedure [10,11], our patients are consequently interested in long-term stable therapeutic results.

To date, there is little evidence that results achieved in the short term after regenerative therapy can be kept stable in the long term (for 10 years or more) [2,11,12,13,14,15,16,17,18]. Since the histological proof of a successful periodontal regeneration is not possible without removing the corresponding tooth from the oral cavity, other, surrogate parameters must be used to prove the success/stability of regenerative procedures. For this reason, the comparison of regenerative treatment with conventional OFD, mentioned above, is often made with regard to attachment level changes [19]. Open-flap debridement provides an epithelial attachment that will be destroyed after transformation into a parakeratinized and microulcerated pocket epithelium by established lesions of gingivitis. Thus, if regeneratively treated sites are as stable as originally periodontally reduced but gingivally healthy or gingivitis sites and, thus, at least as stable as non-surgically treated sites, this proves an achievement of high stability by regenerative treatment [20].

The aim of this retrospective cohort study was to analyze the long-term stability of attachment in IBDs 10 years after regenerative treatment, and to compare it with teeth that did not receive any surgical treatment.

## 2. Materials and Methods

The present study, as part of an ongoing follow-up of regeneratively treated IBDs, is based on a subset reporting 5-year data [16,20], and provides prolonged data for 10 years.

### 2.1. Patients

Patients who received regenerative therapy for IBDs between May 2005 and October 2009 at the Department of Periodontology (Goethe-University Frankfurt am Main) were screened for eligibility.

Inclusion criteria were as follows:

- Age ≥ 18 years;
- Initial diagnosis of moderate or severe periodontitis [21];
- Completion of initial active periodontal therapy (modified full-mouth disinfection [22] (stage I and II) and re-evaluation of non-surgical therapy 3 months later (baseline) [9]);
- Complete set of periapical radiographs or panoramic radiographs from baseline;
- Presence of comprehensive baseline and 12-month data (PPD, CAL, plaque (PlI) [23], and bleeding on probing (BOP)) at 6 sites per tooth;
- At least one regeneratively treated IBD [24] (interproximal angular defect, radiographic infrabony component (INFRA) ≥ 3 mm [9], CAL > 6 mm, and PPD ≥ 6 mm before regenerative treatment);
- Full-mouth plaque score ≤ 30% [25];
- Re-examination 120 ± 12 months after regenerative therapy.

Exclusion criteria were as follows:

- Systemic disease or condition (e.g., heart valve replacement) making antibiotic prophylaxis necessary for clinical measurements that trigger transitory bacteremia (e.g., PPD, CAL);
- Intake of cyclosporin;
- Report of chronic alcohol abuse.

All eligible study participants were contacted consecutively and, if possible, recruited. The study was approved by the Institutional Review Board for Human Studies of Goethe University Frankfurt am Main (approval number 251/10, including amendment of 14 April 2020) and registered at the German register of clinical trials (ID: DRKS00021148). All participants were informed of the risks, benefits, and procedures of the study, and gave written informed consent.

### 2.2. Clinical Measurements

At baseline, 12, and 120 ± 12 months postoperatively, the following clinical parameters were assessed at six sites per tooth: marginal plaque (yes/no [25]) and BOP (yes/no; 20 s after periodontal probing), as well as PPD and CAL to the nearest 0.5 mm with a rigid, millimeter-scale periodontal probe (PCPUNC 15; Hu-Friedy, IL, USA). Reference for CAL measurement was the cementoenamel junction (CEJ) or the restoration margin (RM), if the CEJ had been replaced by restauration [20,26]. All clinical measurements were performed by five periodontists (B.S., T.R., K.N., P.E., and H.P.). All participating examiners had been calibrated by repeated PPD and CAL measurements within two earlier studies [27,28].

### 2.3. Periodontal Surgery

The surgical procedures have been described in detail previously [16,20,26]. It was up to the surgeon to decide whether the specific IBD was treated with a membrane or EMD (enamel matrix derivate; Emdogain, Institut Straumann, Basel, Switzerland), with or without filler (Bio-Oss Collagen, Geistlich, Wolhusen, Switzerland). First, an intracrevicular incision and mucoperiosteal flap preparation were carried out until 5 mm of the bony margin was exposed, allowing for a complete visualization of the IBD [29]. Therefore, a modified or simplified papilla preservation flap was designed to obtain primary wound closure and complete coverage of the membrane [30,31]. Afterwards, granulation tissue was removed, followed by scaling and root planing of the root surfaces. Before application of EMD, filler, and/or membrane, the following intrasurgical parameters were documented to the nearest 0.5 mm: distances of (1) CEJ/RM to alveolar crest and (2) CEJ/RM to the bottom of the bony defect at six sites/tooth, as well as (3) INFRA and the 1-, 2-, and 3-wall components of the defect [7,16,20,32].

After root surface conditioning for 2 min using EDTA (PrefGel, Institut Straumann AG), EMD was applied according to the manufacturer’s instructions, starting from the most apical extension of the defect and continuing coronally [16]. If a membrane was used, an adaptation around the trunk of the root was indicated. All prepared flaps were readapted tension-free. Six examiners who were postgraduate periodontics students or periodontal specialists (Susanne Scharf, Rita Arndt^†^, Martin Wohlfeil, B.S., K.N., P.E.) performed all surgeries.

Fifteen patients of the cohort participated in a placebo-controlled randomized clinical trial (RCT); nine of them received 200 mg of doxycycline once daily for 7 days after surgery [26,33]. Six more participants were also part of the RCT, but were assigned to the placebo group. Postoperatively, all participants rinsed with 0.12% chlorhexidine digluconate solution (ParoEx, Sunstar, France) twice daily for 2 min for 5–7 weeks, and refrained from individual plaque control. During this period, weekly controls, including gentle tooth cleaning, were carried out. If necessary, 400 mg of ibuprofen daily was prescribed. Sutures were removed 1–2 weeks after surgery [16,26]. In the first postoperative year, a quarterly supportive periodontal care (SPC) interval was agreed with the patients. From the second year onwards, the interval was allocated using the periodontal risk assessment [34,35].

### 2.4. *10*-Year Re-Examination

Ten years after regenerative therapy for each included test tooth, a respective control tooth was identified by fulfilling the following criteria [20,36]:

- No vertical bone loss at baseline;
- No surgical treatment;
- Control tooth should be contralateral to the test tooth (i.e., same tooth type (anterior, posterior)).

In addition to aforementioned measurements, the following parameters were assessed:

Gingival bleeding index [37], plaque control record [25], and self-reported smoking status (non-smokers, former smokers (stopped smoking at least 5 years ago), active/current smokers (stopped smoking < 5 years ago or currently smoking)) [34]; initial diagnosis [21] was reclassified to stages using interproximal CAL values from baseline periodontal charts, lost teeth, and complexity, and to grades according to the bone loss age index, smoking and diabetes status [38], and number of SPC appointments, which were taken from the respective patient charts.

### 2.5. Statistical Analysis

The patient was defined as statistical unit. Changes in CAL and PPD from 12 to 120 months were defined as the primary and secondary target variables. After controlling for normal distribution, patients were characterized by frequencies/percentages and/or medians (lower (Q1)/upper (Q3) quartile), while clinical parameters were described as medians (Q1/Q3). CAL stability of test and control teeth was defined as CAL changes of 1 mm or less from 1 to 10 years. Two patient groups were categorized by accordance of CAL stability between test and control teeth: (1) stable = both sites were stable, while (2) unstable = at least one of the two sites was not stable, or the test tooth was lost. Comparisons between both groups were made using Pearson’s correlation coefficient or the chi^2^ test.

Inter-(test, control) and intragroup comparisons (baseline/12/120 ± 12 months) for PPD reduction and CAL gain were made using Friedman and Wilcoxon signed-rank tests. A multivariate analysis of variance (ANOVA) for repeated measurements was calculated (dependent variable: CAL change from 12 to 120 months) considering the following independent variables: baseline age, female gender, initial diagnosis (stage IV periodontitis), baseline smoking status (current smoker), intake of antibiotics, frequency (%) of PPD values > 5 mm at re-examination, and SPC number between 12 and 120 months.

A significance level of 0.05 was assumed. All statistical analyses were performed using statistical software (SPSS Statistics 26; IBM, Chicago, IL, USA).

## 3. Results

### 3.1. Patients

A total of 54 patients were re-examined 120 ± 12 months postoperatively; 5 patients were excluded due to missing data (incomplete intrasurgical parameters). Of the remaining 49 patients, 9 lost the regeneratively treated tooth within the re-examination period of 120 months, and one of them lost it before the first follow-up examination after 12 months. In principle, the regenerative procedure showed a long-term stable attachment with a slight CAL loss (CAL 12–120 months (n = 39): −0.5; 1.5/−1.0 mm; −0.18 ± 1.9 mm) on the remaining 39 teeth. A corresponding control tooth could be identified for 27 out of 49 treated sites.

The median follow-up period was 10.3 years, during which time 20.0 SPC appointments/patient took place. Twenty-six patients were treated with EMD (Emdogain, Institut Straumann), one was treated exclusively with a barrier membrane (1 × bioabsorbable L-lactic-D-lactic-glycolic acid–trimethylenecarbonate barrier (Inion GTR, Inion, Tampere, Finland)), and another was treated with a combination of filler (Bio-Oss Collagen, Geistlich) and EMD. Five test teeth (18.5%) were lost between 12 and 120 months after surgery—none of them before the first follow-up examination after 12 months.

The previously defined control tooth definition was violated in four cases due to different tooth types: #19 (test: 14, control: 22), #35 (test: 45, control: 14), #39 (test: 32, control: 24), and #40 (test: 42, control: 34). None of the control teeth were surgically treated during the follow-up period. Patient- and tooth-related characteristics are depicted in Table 1 and Table 2, respectively.

### 3.2. Clinical Parameters

Regarding plaque accumulation, the analysis failed to show any significant changes. The BOP decreased significantly on test teeth from baseline to 12 months (*p* = 0.026), but otherwise remained stable. At baseline, BOP between test and control teeth differed significantly (*p* = 0.012) (Table 3).

Between baseline and 12 as well as 120 months after surgery, both significant PPD reductions (BL–12 months: −3.0; −4.0/−1.9 mm, −3.09 ± 1.82 mm, *p* < 0.0001; BL–120 months: −3.5; −4.0/−2.4 mm, −3.11 ± 1.82 mm, *p* < 0.0001) and CAL gains (BL–12 months: 2.8; 1.0/4.0 mm, 2.66 ± 1.51 mm, *p* < 0.0001; BL–120 months: 2.5; 0.5/3.6 mm, 2.39 ± 1.86 mm, *p* < 0.0001) were observed for regeneratively treated teeth. The study failed to detect significant PPD (0; −0.3/1.0 mm, −0.02 ± 1.52 mm) and CAL (0.5; −0.5/1.0 mm, 0.27 ± 1.46 mm, *p* = 0.395) changes from 12 to 120 months postoperatively. Intragroup comparisons of the control teeth failed to show significant differences. Between 12 and 120 months, both PPD (*p* = 0.653) and CAL (*p* = 0.320) failed to show significant changes, i.e., remained stable (Table 4).

A total of 15 out of 27 patients (55%) showed agreement regarding long-term CAL stability (*p* = 0.354). The comparison between CAL-stable and CAL-unstable patients showed a significant difference for PRA distribution after 10 years (*p* = 0.037), as well as a tendency towards a lower PCR (*p* = 0.525) and more teeth (*p* = 0.126) after 10 years, as well as more SPC visits (*p* = 0.220) in case of sites with stable CAL. However, group sizes were significantly different (*p* = 0.028) (Table 5).

Multivariate ANOVA for repeated measurements did not show any significant associations between or within patients (Table 6).

## 4. Discussion

A recently published systematic review comparing regenerative and conventional surgical therapy approaches for IBDs concluded that regenerative procedures should be the gold standard for treatment of IBDs ≥ 3 mm [3].

The present study was able to show for regeneratively treated teeth that there are significant gains in clinical attachment 1 year after surgery, which can be kept similarly stable for 10 years as in teeth (controls) that have not received any surgical treatment. While the positive effect of regenerative therapy methods has already been confirmed in several studies over at least 10 years [13,14,15,17,39], a simultaneous long-term comparison with non-regeneratively treated teeth has only been drawn in a few studies [11,12,18,40].

As already mentioned in the introduction, it is not possible to prove the formation of a new connective tissue attachment without histology. Therefore, so-called surrogate parameters (e.g., PPD and CAL) are used, making it possible to derive the clinical benefit of an intervention. Subgingival instrumentation of a root surface usually leads to a reduction in inflammation and an increased tissue resistance against probing, which clinically may be interpreted as a CAL gain, but histologically is probably more likely to represent an epithelial attachment (long junctional epithelium). While epithelial attachment is already destroyed in the established gingivitis lesion, the advanced lesion (i.e., periodontitis) must have been reached in order to destroy the connective tissue attachment [41]. Consequently, connective tissue attachment is considered to be more robust and long-term stable than the epithelial attachment [20]. Assuming that the regeneratively treated sites in the present study would “only” have formed an epithelial attachment similar to that of control teeth instrumented in the context of active periodontal therapy/SPC, the long-term stable result—which is comparable to periodontally reduced but gingivally healthy/gingivitis sites that did not receive any surgical intervention—indicates a high stability achieved by regenerative treatment [20].

Regarding the comparison of regenerative and conventional treatment methods, numerous long-term observations between 10 and 20 years failed to show significant differences between regenerative and conventional treatment groups for the long-term changes in short-term-achieved CAL results (CAL changes over 10 years, test (EMD): −0.5 mm; control (OFD): −0.2 mm [12]; test (bioabsorbable polylactide acetyl tributyl citrate barriers, PLA): 0.65 ± 2.08 mm; control (OFD): −0.05 ± 2.61 mm [40]; CAL changes over 20 years, test (non-resorbable titanium-reinforced expanded polytetrafluoroethylene barriers and non-resorbable expanded polytetrafluoroethylene barriers: 0.1 ± 0.3 mm and 0.5 ± 0.1 mm, respectively; control (OFD): 1.7 ± 0.4 mm [11]; test (bioabsorbable PLA barriers): 0.93 ± 0.66 mm; control: 0.0 ± 2.83 mm [18]). While most of these studies show a slight increase in CAL after regenerative treatment [11,18,40], the present study and the findings of Sculean et al. (2008) show a low, but comparable, attachment loss between 1 and 10 years [12]. This may be because these two studies (almost) exclusively used EMD, whereas the other studies used GTR approaches, and had the same follow-up period. However, the present study was the only one in which the control teeth were treated exclusively non-surgically, and is therefore not comparable to the results of the control group with the aforementioned studies.

Similar to the findings of Cortellini et al. (1996), the present study formed subsamples regarding the agreement of achieved long-term CAL stability on test and control teeth within each patient; due to the lower number of cases in our study, only two groups were formed instead of three [36]. There was 19% less agreement (56%) regarding CAL stability between 12 and 120 months than found by Cortellini et al. (75%) between 12 and 60 months. Therefore, the conclusion stated by Cortellini et al. (which was critically revised in context of 20-year data [11])—that factors influencing both sites within the same patients (i.e., patient-related factors) may have a major impact on long-term stability [36]—is not supported by the present data. Nevertheless, a trend can be seen in patient-related data, as the CAL-stable group tends to contain more non-smokers (CAL-stable: 46.7%; CAL-unstable: 25.0%) and shows better plaque control after 10 years (CAL-stable: 23.0%; CAL-unstable: 33.5%), with the possible consequence of a higher resulting median tooth number (CAL-stable: 27.0; CAL-unstable: 20.5) which, in turn, could possibly be due to more SPC appointments between 12 and 120 months (CAL-stable: 22.0; CAL-unstable: 17.0). The latter may be explained by the significant difference in PRA distribution, with more than twice as many patients at moderate risk in the CAL-stable (86.7%) group compared to the CAL-unstable group (41.7%). However, in terms of regenerative therapy, treatment modalities as well as defect-specific characteristics seem to play an important role, and must be considered in addition to patient-related factors [11,42]. A difference between the two studies is that in the present observation most patients were treated solely by EMD application, whereas Cortellini et al. re-examined guided tissue regeneration procedures using barrier membranes [36,43].

A comparable study did not show any tooth loss when using EMD alone during a 10-year observation period. This may be due to the approximately 1.5 mm lower infra-alveolar defect component (present study: 5.9 ± 2.1 mm; Sculean et al. (2008) [12]: 4.2 ± 1.2 mm) before surgical therapy and/or the significantly lower number of patients (present study: 27; Sculean et al. (2008): 9 [12]) who were re-examined in this study arm [12]. Within the present cohort, it is noticeable that patients who lost their test tooth showed an average 1.0 mm higher CAL after 12 months (CAL, no tooth loss: 6.3 ± 2.1 mm; tooth loss: 7.2 ± 2.0 mm), an approximately 13% higher PCR (PCR, no tooth loss: 29%; tooth loss: 43%), and a lower total number of teeth (median number of teeth, no tooth loss: 24.5; tooth loss: 21.0) at the 10-year follow-up, as well as twice as many smokers at baseline compared to those who did not lose any teeth (no tooth loss: 18.2%; tooth loss: 40%).

Although multivariate analysis failed to show significant associations with potential risk factors, it must be emphasized that some factors strongly influencing long-term CAL stability have been identified and confirmed several times previously. Among these factors, smoking habits, oral hygiene, susceptibility to disease progression, and regular SPC should be mentioned as major determinants [4,12,16,36,39,42,44,45]. It is likely that the overall low number of patients in this study led to the fact that these factors failed to show a significant influence on changes in CAL between 12 and 120 months.

The following limitations must be specified: (1) The retrospective character of this study facilitates a follow-up period of 10 years, but leads to the involvement of many operators and examiners. In addition, the retrospective assignment of the control teeth leads to a bias. (2) The low number of cases, due to the attempt to generate a comparison to some intra-individual control, enables only a limited interpretation of the data. (3) Four out of twenty-seven control teeth were selected in violation of the appropriate definition, which also may cause bias. (4) It must be considered that 10 teeth were lost in the context of longitudinal data analysis, and were therefore not included in the present study.

## 5. Conclusions

- Short-term CAL achieved by regenerative treatment of IBDs exhibited stability comparable to non-surgically treated, periodontally reduced sites over a 10-year period;
- Agreement of 56% between test and control teeth regarding the CAL stability between 12 and 120 months within each patient, indicating the influence of both patient- and treatment-/site-specific factors on the outcome;
- In total, 79.6% of all regeneratively treated teeth could be retained for 10 years.

## Figures and Tables

**Table 1:** Patient characteristics.

Patient Characteristics	Number (%) or Median; Lower/Upper Quartile
Females	16 (59.3)
Age (years)	57.0; 44.0/60.0
Initial diagnosis	
Stage III/Stage IV	23 (85.2)/4 (14.8)
Grade A/B/C	0 (0)/9 (33.3)/18 (66.7)
Smoking status/characteristics at re-examination	
Non-Smokers	10 (37.0)
Former smokers	11 (40.7)
Active/current smokers	6 (22.2)
Pack years	9.5; 3.9/17.5
GBI (%)	3.0; 1.0/4.0
PCR (%)	28.0; 19.0/46.0
BOP (%)	11.4; 6.5/16.7
Periods (years)	
Baseline–120 months	10.3; 9.9/10.3
12–120 months	9.1; 8.9/9.3
Number of teeth	24.0; 20.0/27.0
PPD (%)	
PPD < 4 mm	90.0; 85.4/96.4
PPD 4–5 mm	6.5; 2.9/11.1
PPD > 5 mm	0.7; 0.0/2.3
Number of SPC visits	20.0; 16.0/27.0
Periodontal risk assessment	
Low risk	2 (7.4)
Moderate risk	18 (66.7)
High risk	7 (25.9)

SPC: supportive periodontal care; PPD: periodontal probing depth; GBI: gingival bleeding index; PCR: plaque control record; BOP: bleeding on probing.

**Table 2:** Characteristics of test and control teeth.

	Test						Control
Patient ID	FDI Code	Treatment	Intrasurgical Infrabony Component	3-Wall Component	CAL Gain after 10 Years	CAL Gain between 1 and 10 Years	FDI Code
1	11	EMD	5.00	2.00	3.50	0.50	22
2	13	EMD	6.00	4.00	5.00	0.50	23
9	24	EMD	4.00	0.50	2.50	−0.50	14
10	(44)	EMD	4.50	2.00			35
14	45	GTR (membrane)	5.50	4.00	0.50	−0.50	35
16	43	EMD	3.00	2.00	2.00	0.0	33
19	14	EMD	6.00	3.00	3.00	−1.00	22
20	37	EMD	7.00	5.50	3.00	−1.00	47
21	24	EMD	9.00	5.50	4.00	−1.00	15
24	22	EMD	4.50	1.50	−1.00	−3.00	12
25	43	EMD	8.00	2.00	5.00	0.00	33
27	36	EMD	10.00	3.00	4.00	4.00	46
29	15	EMD	11.00	7.00	3.50	−0.50	25
30	33	EMD + filler	5.00	2.00	0.50	−2.50	22
31	33	EMD	7.00	3.00	6.00	2.00	43
35	(45)	EMD	5.00	2.50			14
39	32	EMD	5.50	3.00	0.0	−0.50	24
40	42	EMD	3.50	2.50	1.50	−0.50	34
41	36	EMD	7.00	4.50	0.0	−1.00	26
44	12	EMD	4.50	3.00	2.50	1.50	33
45	36	EMD	5.00	3.00	2.50	0.50	46
47	46	EMD	3.00	2.50	1.50	−1.00	36
49	(15)	EMD	6.00	2.00			25
50	46	EMD	7.00	1.00	3.00	−1.00	36
51	(45)	EMD	5.50	1.00			34
61	(36)	EMD	7.50	5.50			46
62	45	EMD	4.00	3.00	0.0	−1.00	34

EMD: enamel matrix derivative; membrane: l-lactic-d-lactic-glycolic acid-trimethylenecarbonate membrane; CAL: clinical attachment level; ( ) = tooth was extracted.

**Table 3:** Plaque accumulation and bleeding on probing (number/%).

	Marginal Plaque					Bleeding on Probing				
	Test		Control		*p*-Value	Test		Control		*p*-Value
	n	%	n	%		n	%	n	%	
**Baseline**	11	50.0	11	50.0	1.0	14	63.6	5	22.7	0.012
**1 year**	8	36.4	5	22.7	0.453	6	27.3	4	18.2	0.754
**Change** **BL–1 year**	−3		−6			−8		−1		
***p*-Value**	0.508		0.070			0.026		1.0		
**10 years**	13	59.1	8	36.4	0.125	10	45.5	4	18.2	0.070
**Change** **BL–10 years**	2		3			−4		0		
***p*-Value**	0.754		0.508			0.571		1.0		
**Change** **1–10 years**	4		−2			4		−1		
***p*-Value**	0.227		0.375			0.571		1.0		

n: Number of teeth; BL: baseline.

**Table 4:** Periodontal parameters.

	PPD (mm)					CAL (mm)				
	Test		Control		*p*-Value	Test		Control		*p*-Value
	Median; Lower/Upper Quartile	Mean ± SD	Median; Lower/Upper Quartile	Mean ± SD		Median; Lower/Upper Quartile	Mean ± SD	Median; Lower/Upper Quartile	Mean ± SD	
**BL**	7.0; 6.5/8.3	7.27 ± 1.72	3.0; 2.0/4.0	3.36 ± 1.36	<0.0001	9.0; 7.8/10.5	8.95 ± 2.19	3.5; 2.0/5.3	4.05 ± 1.99	<0.0001
**1 year**	4.0; 3.0/5.0	4.18 ± 1.32	3.0; 2.0/3.3	3.05 ± 0.90	0.004	6.0; 4.9/8.1	6.29 ± 2.07	3.0; 2.0/5.3	3.91 ± 2.07	<0.0001
**Change** **BL–1 year**	−3.0; −4.0/−1.9	−3.09 ± 1.82	0.0; −1.0/0.0	−0.32 ± 0.95	<0.0001	−2.8; −4.0/−1.0	−2.66 ± 1.51	0.0; −1.0/0.0	−0.14 ± 0.83	<0.0001
***p*-Value**	<0.0001		0.131			<0.0001		0.454		
**10 years**	4.0; 3.0/5.0	4.16 ± 1.25	3.0; 2.0/3.5	2.93 ± 1.34	0.010	6.8; 5.4/8.1	6.57 ± 2.06	3.0; 2.0/4.9	4.03 ± 2.62	0.0001
**Change** **BL–10 years**	−3.5; −4.0/−2.4	−3.11 ± 1.82	−0.0; −1.1/0.1	−0.43 ± 1.57	<0.0001	−2.5; −3.6/−0.5	−2.39 ± 1.86	0.0; −1.0/1.0	−0.02 ± 1.95	0.0001
***p*-Value**	<0.0001		0.112			0.0001		0.444		
**Change** **1–10 years**	0.0; −0.3/1.0	−0.02 ± 1.52	0.0; −1.0/0.1	−0.11 ± 1.24	0.653	0.5; −0.5/1.0	0.27 ± 1.46	0.0; −1.0/1.0	0.11 ± 1.50	0.320
***p*-Value**	1.0		0.282			0.395		0.977		

PPD: periodontal probing depths; CAL: clinical attachment level; SD: standard deviation; BL: baseline.

**Table 5:** Patient characteristics according to agreement of CAL stability between test and control teeth in each patient.

Patient Characteristics	CAL Stability between 12 and 120 Months (Number (%) or Median; Lower/Upper Quartile)		*p*-Value
	Stable	Unstable/Tooth Loss	
Total number	15	12	0.028
Females	8 (53.3)	8 (66.7)	0.484
Age (years)	54.0; 36.0/60.0	57.5; 45.5/62.3	0.773
Initial diagnosis			
Stage III/Stage IV	14 (93.3)/1 (6.7)	3 (25.0)/9 (75.0)	0.183
Grade A/B/C	0 (0)/5 (33.3)/10 (66.7)	0 (0)/4 (33.3)/8 (66.7)	1.0
Smoking status/characteristics at re-examination			0.359
Non-Smokers	7 (46.7)	3 (25.0)	
Former smokers	6 (40.0)	5 (41.7)	
Active smokers	2 (13.3)	4 (33.3)	
Pack years	9.0; 3.7/37.8	9.0; 5.0/13.8	0.386
GBI during SPC (%)	2.0; 1.0/4.0	3.5; 1.3/6.5	0.606
PCR during SPC (%)	23.0; 16.0/40.0	33.5; 19.5/46.0	0.525
BOP during SPC (%)	14.1; 6.5/17.3	10.9; 7.7/15.5	0.352
Periods (years)			
Baseline–120 months	10.3; 9.9/10.3	10.1; 9.9/10.4	0.632
12–120 months	9.1; 8.9/9.3	9.0; 8.8/9.4	0.239
Number of teeth at re-examination	27.0; 23.0/28.0	20.5; 19.3/24.0	0.126
Intake of antibiotics	8 (53.3)	3 (25.0)	0.137
PPD (%) at re-examination			
PPD < 4 mm	90.1; 85.4/93.7	88.8; 85.5/96.6	0.464
PPD 4–5 mm	6.5; 2.9/13.2	6.3; 2.5/10.1	0.523
PPD > 5 mm	0.6; 0.0/1.2	2.2; 0.0/5.0	0.385
Number of SPC visits	22.0; 16.0/25.0	17.0; 14.5/33.0	0.220
Periodontal risk assessment at re-examination			0.037
Low risk	0 (0.0)	2 (16.7)	
Moderate risk	13 (86.7)	5 (41.7)	
High risk	2 (13.3)	5 (41.7)	

SPC: supportive periodontal care; PPD: periodontal probing depth; GBI: gingival bleeding index; PCR: plaque control record; BOP: bleeding on probing.

**Table 6:** Univariate repeated measures ANOVA of CAL changes between 12 and 120 months in test and control teeth.

	Degrees of Freedom	F-Ratio	*p*-Value
**Between subjects**			
Age at baseline	1	0.338	0.565
Female gender	1	1.276	0.266
Current smoker at baseline	1	0.504	0.482
Intake of antibiotics	1	0.003	0.958
Number of SPC	1	0.065	0.800
Stage IV periodontitis	1	0.057	0.812
Frequency of PPD > 5 mm	1	0.540	0.467
Error	35		
**Within subjects**			
Regeneration	1	0.452	0.506
Regeneration × age at baseline	1	0.010	0.922
Regeneration × female gender	1	0.005	0.946
Regeneration × current smoker at baseline	1	0.204	0.654
Regeneration × intake of antibiotics	1	0.055	0.815
Regeneration × number of SPC	1	0.045	0.834
Regeneration × stage IV periodontitis	1	0.602	0.443
Regeneration × frequency of PPD > 5 mm	1	0.007	0.932
Error	35		

ANOVA: analysis of variance; CAL: clinical attachment level; SPC: supportive periodontal care; PPD: periodontal probing depth.

## Data Availability

The data presented in this study are available upon reasonable request from the corresponding author.
